# The Role of Liver Imaging in Hereditary Hemorrhagic Telangiectasia

**DOI:** 10.3390/jcm9113750

**Published:** 2020-11-21

**Authors:** Joelle Harwin, Mark D. Sugi, Steven W. Hetts, Miles B. Conrad, Michael A. Ohliger

**Affiliations:** 1 Department of Radiology and Biomedical Imaging, University of California, San Francisco, CA 94143, USA; Joelle.harwin@ucsf.edu (J.H.); mark.sugi@ucsf.edu (M.D.S.); Steven.hetts@ucsf.edu (S.W.H.); miles.conrad@ucsf.edu (M.B.C.); 2 Department of Radiology, Zuckerberg San Francisco General Hospital, San Francisco, CA 94110, USA

**Keywords:** HHT, liver, MRI, ultrasound, AVM, bevacizumab, Osler–Weber–Rendu

## Abstract

Hereditary hemorrhagic telangiectasia (HHT) is an autosomal dominant vascular disorder characterized by spontaneous epistaxis, telangiectasia, and visceral vascular malformations. Hepatic vascular malformations are common, though a minority are symptomatic. Symptoms are dependent on the severity and exact type of shunting caused by the hepatic malformation: Arteriosystemic shunting leads to manifestations of high output cardiac failure, and arterioportal shunting leads to portal hypertension. Radiologic imaging, including ultrasound, computed tomography (CT), and magnetic resonance imaging (MRI), is an important tool for assessing liver involvement. Doppler ultrasonography is the first-line screening modality for HHT-related liver disease, and it has a standardized scale. Imaging can determine whether shunting is principally to the hepatic vein or the portal vein, which can be a key determinant of patients’ symptoms. Liver-related complications can be detected, including manifestations of portal hypertension, focal liver masses as well as ischemic cholangiopathy. Ultrasound and MRI also have the ability to quantify blood flow through the liver, which in the future may be used to determine prognosis and direct antiangiogenic therapy.

## 1. Introduction

Hereditary hemorrhagic telangiectasia (HHT), also called Osler–Weber–Rendu, is an autosomal dominant disorder characterized by arteriovenous malformations (AVMs) throughout the body [1]. The hallmark lesion of HHT is the telangiectasia, a direct connection between the arteriole and venule, bypassing the capillary bed [2]. HHT most commonly involves mutations of two genes: endoglin (*ENG*, on chromosome 9, HHT1) or activin A receptor type II-like 1 (*ACVRL1/ALK1*, on chromosome 12, HHT2). The proteins encoded by *ENG* and *ACVRL1*, endoglin and activin receptor-like kinase 1 (*ALK1*) are endothelial receptors involved in the transforming growth factor beta (TGF-β) signaling pathway [3,4]. The TGF-β signaling pathway, which is involved in angiogenesis and in maintaining vascular integrity, is defective or impaired in patients with HHT [5]. Although mutations in endoglin and *ALK1* are the most common, there are over 600 mutations within at least four different genes which may result in HHT [6,7,8].

The Curaçao diagnostic criteria are used to clinically diagnose HHT [9] and are based upon the presence of four findings: (1) spontaneous and recurrent epistaxis, (2) multiple mucocutaneous telangiectases at characteristic sites, (3) visceral involvement, and (4) a first-degree relative with HHT. Depending on the number of criteria that are met, the diagnosis is categorized as "definite" (3 or more criteria), "suspected" (2 criteria), or "unlikely" (1 criterion). The diagnosis may be confirmed by genetic analysis; however, because all genetic mutations have not been discovered that cause HHT, genetic testing does not exclude the disease if the diagnosis is suspected clinically. Typical sites of involvement of HHT include mucocutaneous telangiectases causing epistaxis and gastrointestinal bleeding, and AVMs in the lungs, brain, and liver.

Liver involvement with HHT is common but usually asymptomatic. Of individuals with HHT, 44-74% have hepatic vascular malformations (VMs), but only 8% show symptoms [1]. Liver involvement occurs most often in females and in patients with the HHT2 genotype, and these patients average 48 years old [1,10]. The most common complications of severe liver involvement are high-output cardiac failure (HOCF) and portal hypertension [11]. Less common complications include ischemic cholangitis and bile duct necrosis [12]. Variations in the clinical presentation of symptomatic patients with hepatic VMs reflect the unique characteristics of the blood supply to the liver as well as the vessels involved: hepatic arteries, veins, and portal veins [12].

The purpose of this review is to discuss the nature of liver involvement with HHT and discuss ways in which imaging can aid in the diagnosis of HHT, its complications, as well as monitoring of therapy.

## 2. Blood Flow in the Liver

**Figure 1:**
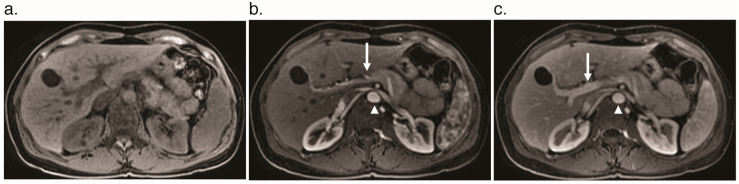
Contrast enhancement phases in a normal liver without vascular shunting on dynamic axial T_1_ fat-saturated magnetic resonance imaging. (**a**) Pre-contrast phase. (**b**) Arterial phase shows enhancement of the aorta (arrowhead) and hepatic artery (arrow). (**c**) Portal venous phase shows enhancement of the portal vein (arrow) and slightly diminished enhancement of the aorta (arrowhead) relative to the arterial phase.

The physiologic manifestations of HHT in the liver reflect the unique nature of its blood supply. The liver receives blood from the hepatic artery and portal vein, with a majority of blood flow (75–80%) normally coming from the portal vein [13]. Blood flows from the hepatic artery through the liver parenchyma, drains into the hepatic vein and inferior vena cava, and then empties into the right heart. The portal vein drains blood from the intestines and empties nutrient-rich blood into the liver, where it then drains to the hepatic vein and into the heart. Distinct contributions from the hepatic artery and portal vein can be seen by imaging at different times following injection of an intravenous contrast agent. The hepatic artery fills with contrast first, followed by the portal vein and then the hepatic veins (Figure 1). Alterations in this timing can signify the presence of a shunt. Hepatic VMs can result in vascular communications between any two of these three vessels (hepatic artery, portal vein, hepatic vein). The physiologic consequences of VMs are ultimately determined by where these abnormal connections occur.

## 3. Hepatic Vascular Malformations

Rather than presenting with single, discrete VMs, liver involvement in HHT is typically diffuse and heterogeneous. Histologically, VMs result in both microscopic changes, such as ectatic sinusoids, and macroscopic vascular anomalies [2]. Diffusely distributed hepatic VMs are relatively characteristic of HHT and unusual in other vascular disorders; therefore, diffuse involvement with VMs should always raise suspicion for underlying HHT [8]. Liver telangiectases are early manifestations of hepatic involvement in HHT [14]. Telangiectases may progress to form more complex vascular malformations and shunts, with up to 21% of patients demonstrating an increase in size and complexity of hepatic VMs after long-term follow-up [4].

Liver vascular shunts are abnormal direct connections where blood bypasses the liver parenchyma. Two major shunt patterns are observed, which lead to different clinical presentations: (1) arteriosystemic (hepatic artery to hepatic vein) and (2) arterioportal (hepatic artery to portal vein) [15]. As mentioned previously, an individual’s ensuing clinical symptoms and course is dependent upon on the type and severity of the hepatic VM. These shunt types may coexist, but clinically, one shunt type often dominates [16]. Certain shunts are more commonly associated with specific complications.

### 3.1. Arteriosystemic Shunt

**Figure 2:**
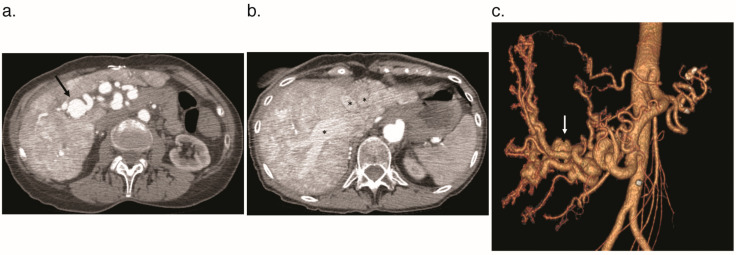
Arteriosystemic shunts on arterial phase CT angiogram of the abdomen in a 73-year-old woman with HHT. (**a**) Marked dilation and tortuosity of the hepatic artery with right hepatic artery aneurysm (arrow). (**b**) Early filling of the hepatic veins (*) on the arterial phase of contrast suggests hepatic arterial to hepatic venous shunting. (**c**) Coronal arterial phase 3D reconstruction demonstrates the marked dilation of the hepatic arteries. The hepatic artery aneurysm is denoted by the arrow.

An arteriosystemic shunt occurs when the hepatic artery is in direct communication with the hepatic vein (Figure 2). This causes arterial blood to bypass the liver parenchyma and capillary bed and drain directly into the systemic venous system and right heart. This results in decreased systemic vascular resistance and increased cardiac preload and stroke volume, the combination of which may cause HOCF [17]. Because HOCF is the most common complication resulting from hepatic VMs, international guidelines recommend obtaining an echocardiogram at the time of hepatic VM diagnosis [10]. These guidelines also recommended further characterization of cardiac and pulmonary pressures with right heart catheterization in individuals with signs or symptoms of HOCF [10]. At cardiac catheterization, patients with HOCF demonstrate increased right atrial, pulmonary wedge, and pulmonary artery pressures [18]. An additional complication evident in HHT patients with HOCF is an increased rate of atrial fibrillation, occurring in approximately 1.6/100 persons [10].

### 3.2. Arterioportal Shunt

**Figure 3:**
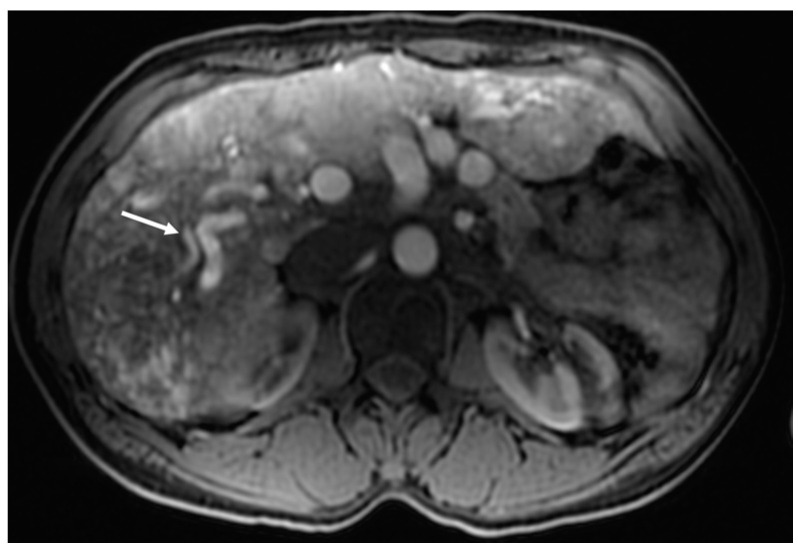
Arterioportal shunt from the hepatic artery to portal vein in a 51-year-old woman with HHT. Axial T_1_ MR image with fat saturation acquired during the arterial phase of contrast shows early filling of a right portal vein branch (arrow) adjacent to the dilated hepatic artery, and associated perfusion anomaly in the right hepatic lobe.

An arterioportal shunt occurs when the hepatic artery is in direct communication with the portal vein (Figure 3). In this type of shunt, the portal vein is exposed to high arterial pressures and also experiences increased blood flow. This leads to presinusoidal portal hypertension, which may lead to the development of ascites, varices, and splenomegaly [12,19].

## 4. Imaging in HHT

Ultrasonography (US), computerized tomography (CT), and magnetic resonance imaging (MRI) each has a role in evaluating the liver in patients with HHT [20]. US is the most common initial screening test, given its broad availability and established grading system [4]. CT and MRI are useful for more targeted evaluations, including for evaluation of focal liver lesions. In addition to targeted evaluations of HHT-related lesions, these examinations are often performed to evaluate suspected abnormalities unrelated to HHT (such as abdominal pain), and therefore understanding the expected imaging findings in patients that HHT is extremely important [21].

### 4.1. Ultrasound

US imaging with grey scale and Doppler evaluation has been a standard screening test for liver AVMs and in the initial work up of patients suspected of having liver involvement due to HHT [10]. US can detect the location of large VMs in the liver, and Doppler imaging permits the evaluation of the direction and magnitude of blood flow [22]. US can also detect focal liver lesions, evaluate the biliary tree, and look for manifestations of portal hypertension such as splenomegaly, ascites, and portal varices.

Dilation of the extrahepatic proper hepatic artery, defined as a diameter greater than 4–5 mm, is one of the earlier manifestations of hepatic VMs [14,15]. This dilated artery can be demonstrated on US. It has been suggested that an additional way to evaluate a dilated proper hepatic artery is by comparing its diameter to that of the splenic artery. Usually, the diameter of the proper hepatic artery is smaller than that of the splenic artery; a reversal of this relationship should raise the suspicion for hepatic VMs [15]. In normal individuals, the diameters of the intrahepatic arteries are usually less than 1.5 mm and can be very challenging to accurately measure on US. With more advanced VMs, dilated intrahepatic arterial branches become more apparent. Another early sign of hepatic VMs is increased velocity within the proper hepatic artery, with some studies suggesting peak flow velocity greater than 100 cm/s as indicative of HHT [15].

**Figure 4:**
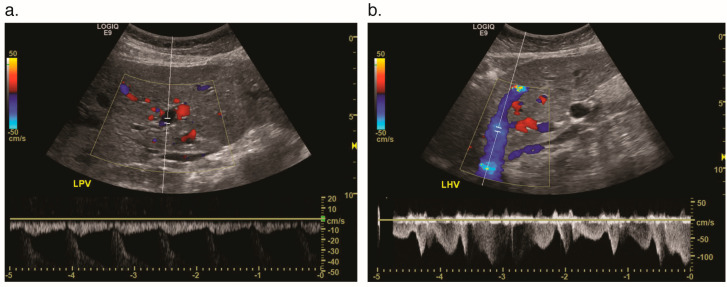
Hepatic vascular malformations on spectral Doppler ultrasound (US) in a 69-year-old woman with HHT. (**a**) Evaluation of the left portal vein shows reversed, pulsatile flow due to arterioportal shunting. (LPV = left portal vein). (**b**) Evaluation of the left hepatic vein shows arterialized waveforms due to artery to hepatic vein (arteriosystemic) shunting. (LHV = left hepatic vein).

On US, arteriosystemic or arterioportal shunts may also be indicated by a low arterial resistive index [14], which is a measure of the end diastolic flow compared to the peak systolic flow. When shunting occurs, the end diastolic flow increases, leading to a diminished resistive index, reflecting a smaller difference between the blood flow in systole and diastole. Another manifestation of arteriosystemic shunts is “arterialization” of the venous waveform, as well as dilation and turbulent flow within the portal and hepatic veins (Figure 4) [14]. In an arterioportal shunt, Doppler US may demonstrate formation of portosystemic collaterals, such as a recanalized paraumbilical vein and even reversal of flow in the portal vein. It is important to note that these findings can also be present in true cirrhosis (e.g., cirrhosis that arises in the setting of viral hepatitis, alcoholic or non-alcoholic fatty liver disease).

Standardized criteria for grading the severity of hepatic VMs based on ultrasound findings has been proposed and demonstrates good interobserver agreement [14,20]. Grading criteria are based upon proper hepatic artery dilation (extrahepatic verses intrahepatic); peak flow velocity; and resistive indices of the proper hepatic artery, degree of hepatic peripheral hypervascularization, and dilation and/or flow abnormalities within the hepatic or portal veins [4]. On Doppler ultrasound, portosystemic shunts may be demonstrated as tubular structures with internal blood flow, communicating between the portal venous branches and hepatic venous branches.

Ultrasound assessment of hepatic VMs can also benefit from injection of intravenous contrast. Contrast-enhanced ultrasound (CEUS) uses microbubbles as intravascular contrast, which are timed to obtain different phases of contrast enhancement such as arterial, parenchymal, and venous [23]. CEUS may offer improved detection of small arteriovenous shunts and can be used to guide real-time intervention [24,25]. CEUS is also becoming increasingly accepted as an effective alternative to multiphase contrast enhanced MR and CT [26]. In HHT, quantitative perfusion imaging with CEUS has been used to identify two distinct subtypes of patients with hepatic VMs [27]. Further research is required to establish the prognostic significance of these groups.

### 4.2. CT and MRI

Although they are not typically first-line screening modalities, CT and MRI can also be used for the evaluation of hepatic VMs. These modalities permit detailed evaluation of vascular anatomy as well as alterations in perfusion that occur with vascular shunting [28,29]. As discussed previously, CT and MRI examinations are typically obtained in different vascular phases following administration of intravenous contrast. These different phases emphasize either hepatic arterial or portal venous blood flow. In a retrospective review of CT scans from 333 patients with HHT acquired over 15 years, 54.1% had liver involvement [21]. Of patients with liver involvement, 47% had telangiectases alone, while the remainder had telangiectases plus large confluent vascular masses, hepatic shunts, or other perfusion abnormalities. CT and MRI are also sensitive to involvement of other organs such as pancreas and gastrointestinal tract (seen in up to 18% of HHT patients in this series).

**Figure 5:**
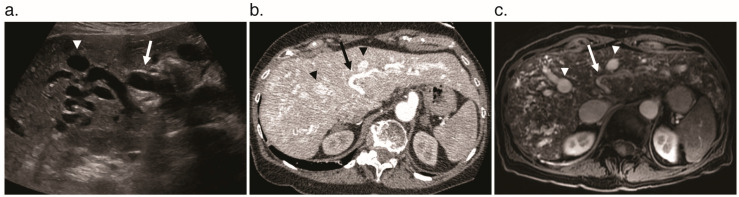
Correlative US, CT, and MR imaging in a 69-year-old woman with HHT. (**a**) Longitudinal grayscale US, (**b**) axial CT angiogram, and (**c**) axial T_1_ fat-saturated gadolinium-enhanced arterial phase images show dilation of the hepatic artery (arrow) and hepatic veins (arrowheads) in this patient with multiple arteriosystemic shunts.

As with ultrasound, CT and MRI can also assess for the size of the hepatic artery and other visceral blood vessels [20]. Early contrast filling of the hepatic vein or portal vein on early (arterial) contrast phases can be evidence of arteriosystemic or arterioportal shunting, respectively (Figure 5) [1]. In addition to the evaluation of vascular shunting, MRI and CT are sensitive to abnormalities of the biliary tree (seen in cholangiopathy) or focal hepatic masses, described in more detail below. Finally, anatomic information obtained using MRI can be complemented by quantitative evaluation of blood flow through the hepatic artery or celiac artery [30]. Another strategy used to evaluate vascular malformations in MRI (though not specific to HHT) is to measure the difference in aortic flow before and after the VM [31].

### 4.3. Catheter Angiography

**Figure 6:**
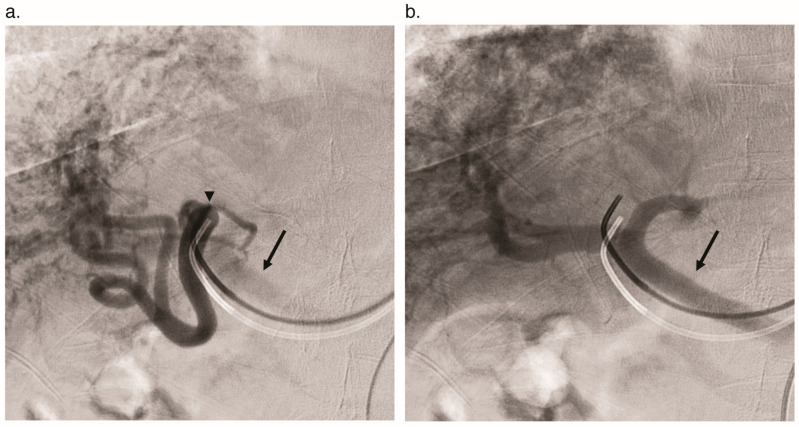
Arterioportal shunt angiography in a 69-year-old woman with HHT. (**a**) Selective catheterization and injection of the hepatic artery (arrowhead) shows dilation of the hepatic arterial system and early contrast opacification of the main portal vein (arrow). (**b**) Portal venous phase shows further opacification of the main portal vein (arrow) and intrahepatic portal veins.

Conventional angiography, with direct arterial visualization, is rarely necessary in evaluating hepatic VMs, given the excellent alternative non-invasive imaging options. However, angiography can be useful in distinguishing between arterioportal, arteriosystemic, and portosystemic shunts (Figure 6). Angiography is often unnecessary because therapeutic embolization of liver AVMs has fallen out of favor due to a high complication rate, described in detail below.

## 5. Treatment of Hepatic Vascular Malformations

Treatment for symptomatic hepatic VMs is based on targeting the clinical symptoms. For example, in an arteriosystemic shunt resulting in HOCF, management is directed towards HOCF with employment of diuretics, salt restriction, etc. [10]. In an arterioportal shunt resulting in portal hypertension, treatment may involve fluid restriction, beta blockers, etc. Of individuals with symptomatic hepatic VMs, 63% have a complete response to first-line treatment [10].

Currently, treatment is not recommended for asymptomatic individuals with hepatic VMs because the majority do not develop complications [10]. Antiangiogenic therapy is being used with increasing frequency for the therapy of HOCF secondary to hepatic VMs. Bevacizumab is a monoclonal antibody targeting circulating vascular endothelial growth factor (VEGF), which stimulates angiogenesis. Intravenous bevacizumab has been shown to decrease cardiac output in persons with symptomatic hepatic VMs resulting in HOCF [32]. However, there is very limited data on the long-term safety of bevazicumab when used for HHT, particularly because periodic maintenance infusions are required [33]. Despite this, bevacizumab is increasingly used as long-term maintenance therapy for HOCF and as a bridge to orthotopic liver transplant for others [6]. It is thought that tyrosine kinase signaling pathways are also involved in angiogenesis. Pazopanib, an oral tyrosine kinase inhibitor most commonly prescribed for the treatment of renal cell carcinoma, has also been shown to potentially reduce bleeding (epistaxis and anemia) in HHT [34] and has also been used for treatment of liver AVMs refractory to bevacizumab.

In severely symptomatic patients who fail first line therapy, more aggressive treatments have been pursued. The curative treatment is orthotopic liver transplant (OLT), which has 82–92% survival rate [10]. With regard to endovascular therapy, hepatic VMs are often considered “do not touch” lesions due to the high risk of mortality and complication. Hepatic artery embolization is associated with a 10% rate of mortality and 20% rate of complication requiring re-intervention [35]. Therefore, such interventions are only recommended in late stage HOCF only when medical therapies fail and OLT is not an option [10].

## 6. Imaging of Liver-Specific Complications on HHT

### 6.1. Pseudocirrhosis

**Figure 7:**
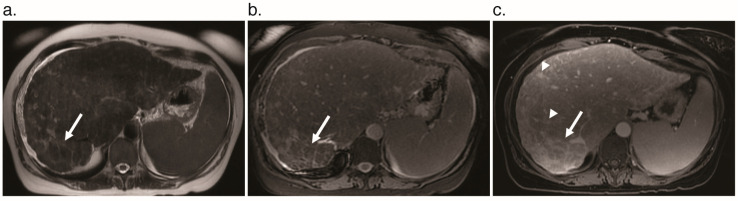
Chronic liver injury with nodular regenerative hyperplasia, pseudocirrhosis, and peripheral fibrosis in a 54-year-old woman with HHT. (**a**) Axial T_1_-weighted MR image shows parenchymal nodularity most prominent in the posterior right hepatic lobe (arrow). (**b**) Axial T_2_-weighted fat-saturated MR image shows a reticular pattern of increased signal in the posterior right hepatic lobe (arrow). (**c**) Delayed enhancement on the axial T_1_ fat-saturated gadolinium-enhanced MR image (arrow), along with multiple telangiectases (arrowheads).

Alterations in liver blood flow caused by VMs in HHT can lead to either diffuse or focal hepatocellular regenerative activity, with fibrosis surrounding the abnormal vasculature [12]. On imaging, hepatic nodules and fibrosis mimic the appearance of cirrhosis. However, in HHT this appearance has been termed ‘‘pseudocirrhosis,’’ as the normal hepatocellular architecture is preserved (Figure 7) [12,16,36]. Unlike in true cirrhosis, there is not the same increased risk of hepatocellular carcinoma (HCC) in pseudocirrhosis.

### 6.2. Focal Nodular Hyperplasia

Focal nodular hyperplasia (FNH) is a common benign liver mass and represents a disorganized proliferation of hepatocytes that can occur in response to VMs. FNH has a 100-fold higher incidence in individuals with HHT [37]. Evaluation with multiphase cross-sectional imaging is usually recommended for evaluation of focal liver lesions. FNH has a highly characteristic imaging appearance on MRI, with early arterial enhancement and a T_2_-hyperintense “central scar” (representing disordered mixture of blood vessels and bile ducts) [29]. It is important for radiologists and clinicians to keep in mind the high incidence of FNH in HHT patients in order to avoid unnecessary biopsies.

**Figure 8:**
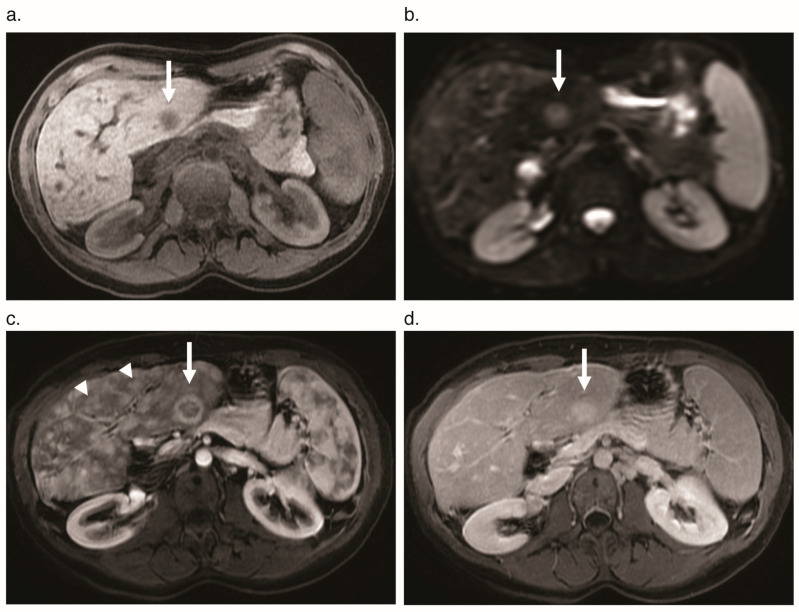
Focal nodular hyperplasia (FNH) mimicking cholangiocarcinoma in a 41-year-old woman with HHT. (**a**) Pre-contrast axial T_1_-weighted MR image shows a focal T_1_ hypointense lesion in segment III of the left hepatic lobe (arrow). (**b**) The lesion shows restricted diffusion at diffusion-weighted MR imaging (arrow). (**c**) Axial T_1_ weighted gadolinium-enhanced MR image with fat saturation shows corresponding peripheral enhancement (arrow) as well as numerous confluent vascular masses (arrowheads). (**d**) The segment III lesion is hyper-enhancing on delayed phase (arrow). The confluent vascular masses are notably isointense to liver parenchyma at this phase. Ultrasound-guided biopsy of the segment III lesion showed features of FNH.

**Figure 9:**
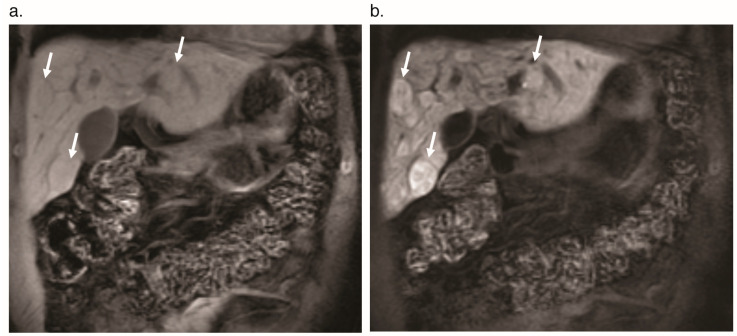
Focal nodular hyperplasia (FNH) in a 61-year-old woman with HHT. (**a**) Numerous hepatic lesions (arrows) are essentially imperceptible given signal characteristics similar to that of background parenchyma on pre-contrast-enhanced coronal T_1_-weighted MR image. (**b**) The lesions (arrows) are hyper-enhancing on the hepatobiliary phase of the gadoxetate-enhanced coronal T_1_-weighted MR image due to expected retention of contrast, compatible with multifocal FNH.

Although FNH is benign, the imaging characteristics can pose a diagnostic dilemma, especially on a background of “pseudocirrhosis” (Figure 8). For example, lesions in HHT patients may not have the T_2_ hyperintense “scar [38]”. In such instances, differentiating benign FNH from malignant HCC (or other tumors) is challenging. One solution is to obtain further imaging with gadoxetate-enhanced MRI. Gadoxetate disodium is a gadolinium-based contrast agent that is actively taken up by hepatocytes. Because FNH is composed of proliferating hepatocytes, gadoxetate is readily taken up by these cells. Most hepatic malignant tumors (such as HCC or metastases) lack the transporter for gadoxetate, and therefore, gadoxetate remains extracellular. This results in unique imaging appearances with FNH retaining contrast (hyperintense) on the delayed “hepatobiliary phase” of gadoxetate-enhanced MRI (Figure 9), while HCC or other genuine neoplasms are usually hypointense relative to surrounding parenchyma. It is so essential to radiologically differentiate FNH from potential HCC due to the differences in management. While suspicious lesions require biopsy, a biopsy is not recommended in FNH on a background of hepatic VMs due to the bleeding risk. 

### 6.3. Cholangiopathy

**Figure 10:**
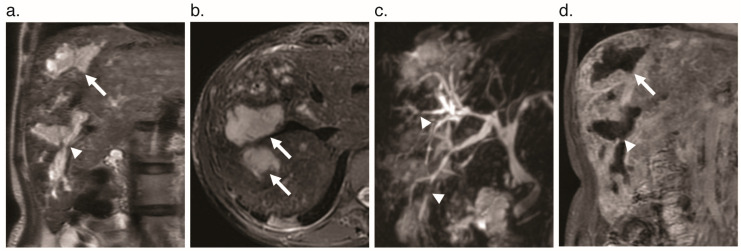
Ischemic cholangiopathy in a 36-year-old woman with HHT. Marked dilation of the intrahepatic bile ducts (arrows) and focal stenoses of the biliary tree (arrowheads) are seen on (**a**) coronal T_2_-weighted SSFSE MR images, (**b**) axial T_2_-weighted SSFSE MR images, (**c**) coronal MRCP maximum-intensity projection image, and (**d**) coronal T_1_-weighted gadolinium-enhanced MR image. (SSFSE = single shot fast spin echo, MRCP = magnetic resonance cholangiopancreatography).

An additional and feared complication of arterioportal or arteriosystemic shunts is cholangiopathy. The hepatic artery is the only blood supply to the bile ducts. Therefore, if blood is shunted from the hepatic artery to the portal or systemic veins, the bile ducts have decreased perfusion. In severe cases, this can result in bile duct ischemia/necrosis, biliary strictures, cholangitis, and even liver abscesses (Figure 10) [10,15].

## 7. Summary and Future Direction

Hepatic VMs are common in patients with HHT, although a majority of them are asymptomatic. Arterial-systemic shunting between the hepatic artery and hepatic vein contributes to HOCF, a major cause of morbidity and mortality for HHT patients. Arterial-portal shunting can lead to portal hypertension. Liver-specific abnormalities include pseudocirrhosis, FNH, and ischemic cholangiopathy.

Non-invasive imaging with US, CT, and MRI plays a valuable role in assessing the hepatic manifestations of HHT. US is the preferred initial screening modality for liver VMs. All three methods can be useful for detecting and distinguishing the type of shunting that is present as well as for the detection of focal lesions as well as the physiologic consequences of portal hypertension.

Because the severity and type of liver shunting is an important determinant of the clinical presentation, particularly the development of HOCF, there is considerable interest in determining whether imaging assessment of hepatic VMs can help predict treatment response in patients undergoing anti-angiogenic therapy. As described above, quantitative assessment of flow and vascular morphology using US or MRI has the potential to help understand the role of the liver in determining the clinical response to these novel therapies.
